# Incorporating Voluntary Medical Male Circumcision Into Traditional Circumcision Contexts: Experiences of a Local Consortium in Zimbabwe Collaborating With an Ethnic Group

**DOI:** 10.9745/GHSP-D-18-00352

**Published:** 2019-03-22

**Authors:** Joseph Hove, Lewis Masimba, Vernon Murenje, Simon Nyadundu, Brian Musayerenge, Sinokuthemba Xaba, Brian Nachipo, Vuyelwa Chitimbire, Batsirai Makunike, Marrianne Holec, Takarubuda Chinyoka, John Mandisarisa, Shirish Balachandra, Mufuta Tshimanga, Scott Barnhart, Caryl Feldacker

**Affiliations:** a Zimbabwe Association of Church-Related Hospitals (ZACH), Harare, Zimbabwe.; b International Training and Education Center for Health (I-TECH), Harare, Zimbabwe.; c Ministry of Health and Child Care, Harare, Zimbabwe.; d International Training and Education Center for Health (I-TECH), Seattle, WA USA.; e Representative of the VaRemba ethnic group, Harare, Zimbabwe.; f U.S. Centers for Disease Control and Prevention, Harare, Zimbabwe.; g Community Health Intervention Project (ZiCHIRe), Harare, Zimbabwe.; h Department of Global Health, University of Washington, Seattle, WA, USA.; i Department of Medicine, University of Washington, Seattle, WA, USA.

## Abstract

Employing voluntary medical male circumcision (VMMC) within traditional settings may increase patient safety and help scale up male circumcision efforts in sub-Saharan Africa. In Zimbabwe, the VaRemba are among the few ethnic groups that practice traditional male circumcision, often in suboptimal hygienic environments. ZAZIC, a local consortium, and the Zimbabwe Ministry of Health and Child Care (MoHCC) established a successful, culturally sensitive partnership with the VaRemba to provide safe, standardized male circumcision procedures and reduce adverse events (AEs) during traditional male circumcision initiation camps. The foundation for the VaRemba Camp Collaborative (VCC) was established over a 4-year period, between 2013 and 2017, with support from a wide group of stakeholders. Initially, ZAZIC supported VaRemba traditional male circumcisions by providing key commodities and transport to help ensure patient safety. Subsequently, 2 male VaRemba nurses were trained in VMMC according to national MoHCC guidelines to enable medical male circumcision within the camp. To increase awareness and uptake of VMMC at the upcoming August–September 2017 camp, ZAZIC then worked closely with a trained team of circumcised VaRemba men to create demand for VMMC. Non-VaRemba ZAZIC doctors were granted permission by VaRemba leaders to provide oversight of VMMC procedures and postoperative treatment for all moderate and severe AEs within the camp setting. Of 672 male camp residents ages 10 and older, 657 (98%) chose VMMC. Only 3 (0.5%) moderate infections occurred among VMMC clients; all were promptly treated and healed well. Although the successful collaboration required many years of investment to build trust with community leaders and members, it ultimately resulted in a successful model that paired traditional circumcision practices with modern VMMC, suggesting potential for replicability in other similar sub-Saharan African communities.

## Abstract (precis)

The successful collaboration resulted in a male circumcision camp where 98% of the 672 boys and men ages 10 and up chose voluntary medical male circumcision (VMMC) while traditional practices were respected. Such collaborations may improve patient safety and increase VMMC uptake in sub-Saharan Africa.

## BACKGROUND

Evidence suggests that offering voluntary medical male circumcision (VMMC) within traditional settings could facilitate safer outcomes for traditional circumcision practices and increase male circumcision coverage.^1^^–^^7^ In Zimbabwe, the majority of people do not belong to ethnic groups that traditionally circumcise.^8^ The few ethnic groups that do practice traditional male circumcision consist of the VaRemba tribe, a group of about 80,000^9^ with ties to Judaism who are concentrated in the Mberengwa region of Midlands Province,^10^ along with the Xhosa and Tshangani.^11^

Among the VaRemba, initiation camps are held every 1 to 2 years to complete the rites of passage from boyhood to manhood. In these camps, experienced traditional circumcisers typically perform male circumcision with the help of the community elders.^12^ The initiation camp is traditionally held in the winter after the harvest season when the community can ensure enough food for the gathering. Winter season camps also coincide with school holidays, making the timing convenient for the many new school-age initiates. Although joining the camp is voluntary, adherence to tradition compels most boys and men to join. Some older VaRemba men in the villages join the camp because they have not yet been circumcised or, conversely, had been circumcised medically but wish to participate in other camp rituals. VaRemba work hard to preserve their cultural traditions and protect communal secrecy surrounding VaRemba initiation rites. Therefore, few additional details are known about the camp activities.

Although traditional male circumcision procedures are poorly documented in Zimbabwe and elsewhere, previous research has found that over one-third of males may experience adverse events (AEs) in traditional settings.^7^ Lack of proper hygiene for both the procedure and the camp environment may contribute to increased AEs in these settings. Restriction of VaRemba camp attendance to only male members of the VaRemba tribe reduces documentation of these male circumcision procedures as well as identification, treatment, and verification of associated AEs.

ZAZIC is a consortium led by the University of Washington's International Training and Education Center for Health (I-TECH) and local implementing partners Zimbabwe Association of Church-Related Hospitals (ZACH) and Zimbabwe Community Health Intervention Research (ZiCHIRe) Project. The consortium receives support from the U.S President's Emergency Plan for AIDS Relief (PEPFAR) and the U.S. Centers for Disease Control and Prevention (CDC) to implement a male circumcision program together with the Zimbabwe Ministry of Health and Child Care (MoHCC). ZAZIC completed nearly 200,000 male circumcision procedures from 2013 to 2017,^13^ with a reported AE rate of 0.3%. Reported AEs may be moderate (requiring intervention or medication) or severe (requiring surgical intervention or hospitalization) in nature. Most of the AEs were infections among younger boys who are more likely to experience AEs than their older peers.^14^

ZAZIC aimed to facilitate and foster a win-win relationship between VaRemba and the MoHCC. The consortium aimed to maintain VaRemba traditional male circumcision ceremonies but with MoHCC-trained, VaRemba health care workers performing the actual male circumcision procedure, according to national male circumcision guidelines. Culturally appropriate education and mobilization efforts would facilitate success,^12^^,^^15^ taking care to ensure active VaRemba participation and leadership at all stages of planning, implementation, and dissemination.^16^^–^^19^ At the core, rather than demanding modern VMMC, this collaborative approach would promote cultural competency and sensitivity among MoHCC and ZAZIC partners^12^ with the aim of forming a long-term, mutually beneficial partnership focused on future VMMC scale-up in the area. The resulting VaRemba Camp Collaborative (VCC) culminated from years of sensitization, communication, and relationship building. This article aims to provide details on the pathway to the VCC's ultimate success, along with data on uptake of VMMC, to encourage and inform similar partnerships in the future.

## PROGRAM DESCRIPTION

### Laying the Foundation

Working closely with the Provincial Medical Director, the District Administrator, and the Principal Cultural Heritage Officer, ZAZIC sought to establish a partnership with the VaRemba community to provide safe, standardized male circumcision and reduce AEs during traditional initiation rites. Initial VCC discussions began in 2013 with a 3-day meeting between ZAZIC, the MoHCC, the District Health Executive, and the VaRemba chief and elders, resulting in a formal declaration of collaboration. Subsequently, ZAZIC took steps to increase its presence in the local area and provide additional support for VaRemba traditional male circumcision. VaRemba cultural practice mandates that only VaRemba males be allowed in initiation camps and that VaRemba elders select the camp location. However, ZAZIC team members were aware of several hygiene concerns: no shoes are allowed in the camps; most participants did not have underwear; there were no toilet facilities; and male circumcision recovery took place in self-constructed shelters made predominantly from plastic sheeting and branches.

There were several hygiene concerns with the traditional male circumcision camps, including the self-constructed recovery shelters made predominantly from plastic sheeting and branches.

**Figure:**
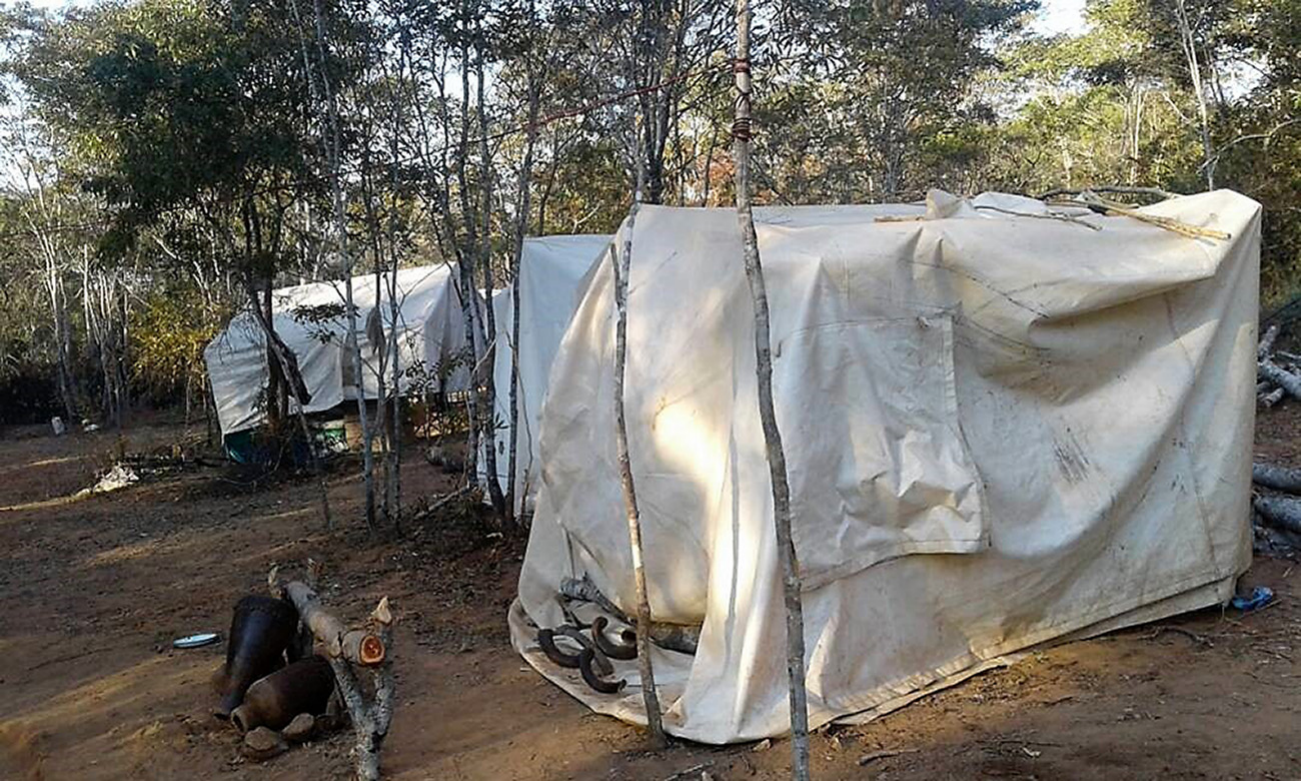
Partial view of male circumcision camp shelters in Zimbabwe. © 2018 Joseph Hove/Zimbabwe Association of Church-Related Hospitals (ZACH)

With this knowledge, between 2013 and 2016, the VCC supported the VaRemba initiation camps in several ways. First, the district supplied the VaRemba with medical male circumcision kits (i.e., surgical supplies) to augment the safety of traditional male circumcision procedures conducted by VaRemba circumcisers. ZAZIC also provided commodities for wound care, supplemental nutritional support, a generator, and tents to help ensure safer procedures. Lastly, ZAZIC provided male circumcision teams a vehicle to transport clients for the procedure and medical care, if needed. Between 2013 and 2016, camp enrollment reduced by more than half: 533 in 2013; 301 in 2014; and 227 in 2016. These enrollment declines were likely due to lack of guaranteed food in camp; simultaneous camps by other VaRemba groups; and some VaRemba opting into VMMC at the hospital rather than at the camp.

During the 2013–2016 period, several key steps helped solidify the VCC partnership. First, strategic ZAZIC staff, including demand creation and clinical teams, paired with MoHCC VMMC officers (Provincial VMMC Officer, District AIDS Council Officer, District Administrator's Arts and Cultural Officer) maintained close contact with VaRemba leaders, meeting several times per year for both formal and informal conversations. These meetings often overlapped with scheduled ZAZIC support visits to area hospitals in the districts and helped build trust within the partnership. Between meetings, VaRemba leaders and clinicians continued to communicate frequently by phone with ZAZIC clinicians, and ZAZIC doctors provided medical support and emergency care for traditional male circumcision clients when requested.

In late 2016, 2 male VaRemba nurses were trained in VMMC according to MoHCC guidelines in response to the requirement that only VaRemba members attend the initiation camps. These nurses were also trained in MoHCC documentation tools for VMMC. ZAZIC also began working closely with a trained demand creation team comprised of circumcised VaRemba men to encourage uptake for VMMC. Although the VaRemba community has its own communication channels through which they mobilize for the initiation camps, ZAZIC took advantage of VaRemba advocacy efforts to further advertise the camps and, simultaneously, promote other VMMC services for VaRemba and non-VaRemba men and boys in health facilities across the district. These additional activities strengthened the VCC, building confidence in the good intentions and follow-through of partners on all sides.

Because only VaRemba could attend the male circumcision camps, 2 VaRemba nurses were trained in VMMC.

### Planning the VaRemba Camp Collaborative

In June 2017, a meeting was held with 14 VaRemba community leaders, the District Administrator, the National AIDS Council, ZAZIC, and the MoHCC, represented by the Provincial Medical Director, the National Male Circumcision Coordinator, and Mberengwa district health officials. Following this meeting, the VaRemba formally adopted national VMMC guidelines for their upcoming initiation camp, including informed consent, counseling, and HIV testing. VaRemba leaders also granted permission for non-VaRemba, ZAZIC medical doctors to reside in the camp to attend to any potential male circumcision complication or emergency—a critical concession for increased safety. ZAZIC supported mobilization of males over age 10 in the area, promoting the option of modern VMMC at the camp (for VaRemba males only) or routine VMMC at the local hospital (for VaRemba or non-VaRemba males). Mobilizers also emphasized screening of other health conditions that accompanied male circumcision, as per routine practice. ZAZIC, in coordination with the district, also provided male circumcision kits, commodities, salt, and underwear. The National AIDS Council provided supplemental food for the duration of camp.

### Camp Implementation

The camp was conducted from August 1 to September 6, 2017, in a dusty, mountainous location. The camp was located 26 km from a hospital and 4 km from a clinic. VMMC was offered to males above 10 years of age according to MoHCC policy. Informed consent followed MoHCC policy for adults and youth. For youth, specifically, informed consent also included additional steps. First, most clients were mobilized before coming into the camp, and their consent forms were witnessed by the mobilizer and signed by parents at home weeks before camp. Second, the program-associated drivers (demand creation specialists and program managers) collected many camp attendees from their homes and further verified consent with the parents/guardians at that time. Clients brought to camp by their parents/guardians signed informed consent at reception. For all clients, VaRemba nurses completing the male circumcision procedure verified and signed every informed consent form.

All routine MoHCC data for each VMMC procedure completed was recorded in the MoHCC VMMC register and client intake forms by VaRemba nurses. Routine data included documentation of demographic data, eligibility, informed consent, the procedure, and postoperative clinical review, including intraoperative and postoperative AEs. VMMC counseling, HIV testing, and the male circumcision procedure were done on day of arrival. Clients joined and were circumcised throughout the camp period, ensuring a constant flow of clients.

Transportation was provided for hospital staff and clients; dedicated vehicles also ensured a consistent supply of available commodities to match client flow. Tents and couches supplied by ZAZIC served as an outreach-based medical facility. The MoHCC-trained VaRemba nurses performed and documented VMMCs within the camp setting and managed mild AEs among traditional male circumcision clients. The 2 non-VaRemba ZAZIC medical doctors, under strict confidentiality agreements, provided oversight as well as treatment for all moderate and severe AEs. Additional medical supplies, transportation, and inpatient care, if needed, were available from local health facilities. Cost reimbursements were given according to the structure provided by MoHCC performance-based financing (PBF) scheme for VMMC,^20^ including specific allocations for medical circumcisers, local male circumcision demand creation mobilizers, and temporary demand creation mobilizers (VaRembas recruited from the community specifically for the camp). VaRemba leaders, as a group, were also all paid as part of male circumcision routine service delivery for providing the outreach location (mobile camp).

## METHODS

As per routine MoHCC VMMC data collection procedures, VaRemba nurses collected data on all VMMC clients on 2 forms: the client intake form and the VMMC register. Details on these data collection procedures are provided elsewhere.^21^^,^^22^ In brief, the client intake form is the primary tool used by VMMC service providers to document detailed client information and includes data on demographics, vital signs, HIV testing, pre-procedure indicators, eligibility, consent verification, procedure details, AEs, and post-procedure follow-up. Each client has a unique client intake form. Information contained in the VMMC register also includes demographic information, male circumcision type (surgical, either dorsal slit or forceps-guided, or by device), AEs, and follow-up visit adherence. Each VMMC client is documented in one row of the program register. Each register page includes a summary line of aggregate client procedures, AEs, and follow-up visits. Register data are used to complete the monthly return form with aggregate reporting indicators including number of VMMCs by age, HIV status, VMMC type, AE by severity, and at least 1 follow-up visit within 14 days of procedure. VMMC data from camp attendees were included as part of Mberengwa District Hospital outreach statistics and reported to both ZAZIC and the MoHCC as part of the routine monthly return form. Descriptive statistics were compiled from the client intake forms and VMMC register used during camp.

## RESULTS

Of 672 males ages 10 and older who attended the initiation camp, provided informed consent, and were screened for male circumcision, all were found eligible and without contraindications. In total, 657 (98%) chose VMMC. In accordance with MoHCC policy, dorsal slit VMMC method was used for all 225 boys ages 10–14 (34%) while forceps-guided VMMC was used for the 432 (66%) boys ages 15 and older (Table). All men were tested for HIV as part of routine VMMC; 4 clients tested HIV-positive and underwent VMMC. Of the 4 testing positive for HIV, 1 of the clients was already on antiretroviral therapy and the other 3 were linked with care. Having the support of VaRemba leaders was critical for VMMC acceptance. Due to their support for, and approval of, the medical circumcision performed by the VaRemba nurses, almost all eligible boys and men opted for modern VMMC, suggesting little reticence to accept modern circumcision within the camp context.

98% of the males ages 10 and older who attended the 2017 camp chose VMMC.

**TABLE.** Key Voluntary Medical Male Circumcision Characteristics and Outcomes From Varemba Camp, Zimbabwe, August 1, 2017–September 6, 2017 (N=657)

	Age Group						
	10–14	15–19	20–24	25–29	30–49	50+	Total
**HIV status, No. (%)**							
HIV-negative	224 (34.1)	148 (22.5)	118 (18.0)	73 (11.1)	89 (13.5)	1 (0.2)	653 (99.4)
HIV-positive	1 (0.2)	0 (0.0)	0 (0.0)	0 (0.0)	3 (0.5)	0 (0.0)	4 (0.6)
Unknown	0 (0.0)	0 (0.0)	0 (0.0)	0 (0.0)	0 (0.0)	0 (0.0)	0 (0.0)
**Male circumcision method, No. (%)**							
Dorsal slit	225 (34.2)	0 (0.0)	0 (0.0)	0 (0.0)	0 (0.0)	0 (0.0)	225 (34.2)
Forceps-guided	0 (0.0)	148 (22.5)	118 (18.0)	73 (11.1)	92 (14.2)	1 (0.2)	432 (65.7)
**Moderate or severe adverse events, No. (%)**	1 (0.2)	0 (0.0)	2 (0.3)	0 (0.0)	0 (0.0)	0 (0.0)	3 (0.5)
**Clients with ≥1 postoperative visit within 14 days of procedure, No. (%)**	225 (34.2)	148 (22.5)	118 (18.0)	73 (11.1)	92 (14.2)	1 (0.2)	657 (100.0)
**Total, No. (%)**	225 (34.2)	148 (22.5)	118 (18.0)	73 (11.1)	92 (14.2)	1 (0.2)	657 (100.0)

In total, 68 traditional male circumcision procedures were performed in the camp. Although underage boys were offered deferment of VMMC for a future camp, 53 boys under age 10 (who were ineligible for VMMC) were circumcised using traditional methods and 15 adult men also chose traditional male circumcision. Due to the presence of VaRemba nurses in the camp, traditional male circumcision procedures also benefited from improved hygiene. Traditional VaRemba circumcisers tried to follow the clinical way of thoroughly cleaning the penis, washing hands, and putting on new gloves for each male circumcision. They also used a new razor blade for each client and an antiseptic (methylated spirits) for pre-procedure cleaning.

Hygienic conditions of the outdoor camp setting and follow-up recovery were suboptimal. Therefore, several infection prevention steps were implemented before, during, and after the procedure. First, temporary toilet facilities were erected, and campers were encouraged to bathe in a nearby river with soap provided by ZAZIC before the circumcision procedure. Second, VaRemba nurses adhered to all intraoperative hygienic practices as recommended by the MoHCC. Third, as most initiates were young boys ages 10–14, ages that are at greater risk of infection than older clients,^14^ all younger clients, including those circumcised traditionally, were given a stat dose (within 1 hour of the procedure) of amoxicillin (250 mg or 500 mg, depending on weight) for infection prophylaxis. Adults who showed signs of poor hygiene, identified at the discretion of the VMMC nurses, were also administered the same broad spectrum antibiotic. Amoxicillin was chosen because it is the most readily available and affordable broad-spectrum antibiotic. Before administration, all were asked about allergies per MoHCC preoperative assessment. Fourth, VaRemba nurses were residents in the camp and conducted daily wound inspection for all male circumcision patients from Day 2 bandage removal and onward. This vigilance helped identify AEs, such as infections, early and likely reduced moderate or severe complications. Lastly, all camp attendees under 15 years of age were provided with clean underwear.

**Figure:**
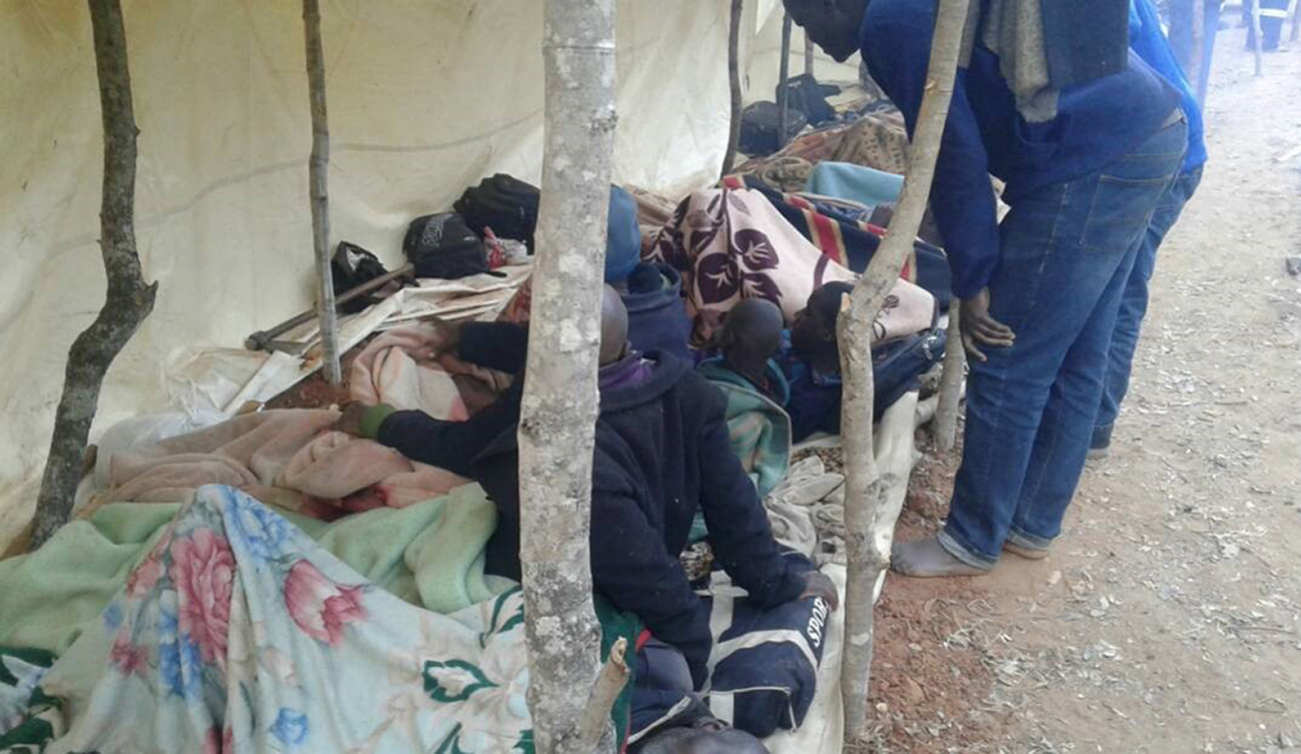
Adult VaRemba clients receiving routine postoperative review by VaRemba nurses in Zimbabwe. © 2018 Joseph Hove/Zimbabwe Association of Church-Related Hospitals (ZACH)

Only 3 moderate infections (0.5%) occurred among the VMMC cases; all were promptly treated with antibiotics (Cloxacillin) and healed well while still in camp. There were 7 mild infections, 4 of them among those traditionally circumcised. With daily reviews and timely appropriate management, these mild AEs did not progress into moderate or severe infections. Mild AEs are not reported per MoHCC guidelines. Traditional circumcision clients received the same follow-up care as those circumcised medically; however, the 53 traditional procedures were not recorded on MoHCC forms.

Only 3 moderate infections occurred among the VMMC cases.

Additional details about the camp context are not shared to protect the privacy of VaRemba collaborators, respect cultural sensitivity, and maintain trust within the VCC.

## DISCUSSION

The VCC is one of the few of its kind in Zimbabwe or elsewhere to successfully pair traditional circumcision practices with modern VMMC. In 2017, 725 men attended the VaRemba initiation camp, reversing the progressive annual drop in attendance since 2013, and 98% of eligible men chose VMMC over traditional circumcision. Multiple factors might explain the VaRemba's willingness to collaborate with the ZAZIC consortium and the MoHCC. First, ZAZIC demand creation teams are from the communities in which they serve. Therefore, they were likely more successful in correctly addressing myths and misconceptions about medical circumcision through various community engagement strategies, including school visits, soccer and music galas, and community dialogue. Also, HIV prevalence in Midlands province, where Mberengwa is located, is 14%.^23^ ZAZIC teams made formal presentations to VaRemba elders on basic HIV/AIDS facts and the preventive role of VMMC to raise awareness about the national HIV prevention program and its goals. These activities were consistently complemented with a clear ZAZIC emphasis promoting respect for both traditional circumcisers and cultural practice.^24^ The VCC goal was a partnership where traditional and medical practice could come together with the medical practitioners offering the medical procedure and follow-up only while all other camp activities would remain the same. The availability of food provided by ZAZIC and the National AIDS Council also likely increased participation. Lastly, parents may have been more confident in sending their children to the camp with the knowledge that modern VMMC would be provided, similar to the type of procedure routinely delivered in local health centers.

The goal of the collaborative partnership was to marry traditional and medical male circumcision practices to improve safety while respecting tradition.

To maintain the low AE rates observed in this collaborative effort, more research on use of prophylactics within the VMMC context is warranted. ZAZIC's program prioritizes VMMC quality in both outreach and static sites. ZAZIC also emphasizes messages, policies, and practices that promote hygiene, cleanliness, and proper wound care before, during, and after VMMC. Although use of surgical antibiotic prophylaxis is recommended by the World Health Organization's (WHO's) Surgical Safety Checklist,^25^ WHO does not recommended antibiotic prophylaxis for VMMC.^26^ In Zimbabwe, use of prophylactic antibiotics is not part of either ZAZIC or MoHCC standard VMMC program practice. However, it is possible that the antibiotic protocol used within the traditional setting may have contributed to the low postoperative infection rates at the camp.^27^^–^^29^ Use of a different prophylactic antibiotic (Flucloxacillin) did not show a positive effect to prevent postoperative infections after male circumcision in a large VMMC program in South Africa.^30^ A smaller study comparing male circumcision methods in Mozambique employed prophylactic antibiotics in response to perceived higher risk for wound infection.^31^ Although the study was not aimed at assessing the influence of antibiotic prophylaxis, it did find a reduction, albeit non-significant, in postoperative infection from 6.9% to 1.4% after mid-study initiation of Cloxacillin prophylaxis.^31^ Additional study of the use of antibiotic prophylaxis in specific contexts may be helpful to inform future VMMC policy.

Lastly, although this collaboration was positive, there are several lessons learned from the formation and implementation of the VCC that inform subsequent camp implementations. First, to avoid complications applying the MoHCC PBF model in the current Zimbabwean VMMC program, clear protocols are needed to guide acquisition and disbursement of funds, food, and transportation. Elders were not considered in MoHCC PBF guidelines; therefore, the VCC negotiated to pay these VaRemba leaders as mobilizers, providing the same amount per client as other mobilizers. Second, the VCC took time and effort above that needed for routine VMMC; however, consideration of more remote communities and settings merits attention. For example, in response to the VaRemba preference for only trained VaRemba clinicians in the camp, the VCC was less effective in early years while VaRemba nurses underwent formal MoHCC VMMC training. Although this caused delays, this training was ultimately critical for success by increasing both sustainability of the collaboration and VaRemba ownership of the VMMC process. Moreover, communication challenges must be addressed with care. In Zimbabwe, the VaRemba are fragmented into smaller groups with separate leadership, complicating efforts to establish clear, appropriate, and consistent lines of communication and joint leadership. Difficulties created by community relationships, remote locations, and poor mobile phone networks reinforce the VCC emphasis on close communication between all partners and stakeholders to help ensure proper planning, preparation, and implementation in the future. Lastly, additional preparation is needed in the more complex camp setting, especially for younger males, to ensure proper informed consent, counseling, and follow-up. The resident MoHCC-trained VaRemba nurses, in close cooperation with the ZAZIC medical doctors, worked throughout the camp implementation period to ensure verification of documentation at all points of client entry, implementation of comprehensive male circumcision counseling in the local language (including HIV counseling), and maintenance of hygienic standards throughout the postoperative period in accordance with MoHCC guidelines.

## CONCLUSIONS

The VCC resulted from 4 years of persistent consultation and collaboration between ZAZIC, the MoHCC, and VaRemba traditional leaders. Multiple stakeholders were involved and informed at all stages of the collaboration, from inception to planning for replication. The approach undertaken to provide VMMC in traditional male circumcision camps appears to be both safe and acceptable for the initiates, as demonstrated in the 2017 camp effort. After the success of this effort, the VCC will continue implementation of the partnership model for future initiation camps and other VaRemba groups are now requesting similar collaborations. In fact, in 2018 ZAZIC began working with 6 VaRemba communities in other geographic areas in Zimbabwe, culminating in 1 additional successful camp collaboration in Gokwe South District: 206 medical VMMC procedures were performed without complication. Capitalizing on the momentum of the VCC, ZAZIC is also actively working to identify other VaRemba health care workers across Zimbabwe for MoHCC VMMC training. This partnership model may be replicable in other contexts in Zimbabwe and the region, expanding efforts to provide VMMC within traditional settings.

## Notes

**Peer Reviewed**

**Funding:** The male circumcision program on which this article is based is supported by the U.S. President's Emergency Plan for AIDS Relief (PEPFAR) through the U.S. Centers for Disease Control and Prevention (CDC), under the terms of CDC cooperative agreement 5U2GGH000972 to I-TECH.
